# Robust colour constancy in red-green dichromats

**DOI:** 10.1371/journal.pone.0180310

**Published:** 2017-06-29

**Authors:** Leticia Álvaro, João M. M. Linhares, Humberto Moreira, Julio Lillo, Sérgio M. C. Nascimento

**Affiliations:** 1 Anglia Vision Research, Department of Vision and Hearing Sciences, Anglia Ruskin University, Cambridge, United Kingdom; 2 Facultad de Psicología, Universidad Complutense de Madrid, Pozuelo de Alarcón, España; 3 Centre of Physics, University of Minho, Braga, Portugal; University of Sussex, UNITED KINGDOM

## Abstract

Colour discrimination has been widely studied in red-green (R-G) dichromats but the extent to which their colour constancy is affected remains unclear. This work estimated the extent of colour constancy for four normal trichromatic observers and seven R-G dichromats when viewing natural scenes under simulated daylight illuminants. Hyperspectral imaging data from natural scenes were used to generate the stimuli on a calibrated CRT display. In experiment 1, observers viewed a reference scene illuminated by daylight with a correlated colour temperature (CCT) of 6700K; observers then viewed sequentially two versions of the same scene, one illuminated by either a higher or lower CCT (condition 1, pure CCT change with constant luminance) or a higher or lower average luminance (condition 2, pure luminance change with a constant CCT). The observers’ task was to identify the version of the scene that looked different from the reference scene. Thresholds for detecting a pure CCT change or a pure luminance change were estimated, and it was found that those for R-G dichromats were marginally higher than for normal trichromats regarding CCT. In experiment 2, observers viewed sequentially a reference scene and a comparison scene with a CCT change or a luminance change above threshold for each observer. The observers’ task was to identify whether or not the change was an intensity change. No significant differences were found between the responses of normal trichromats and dichromats. These data suggest robust colour constancy mechanisms along daylight locus in R-G dichromacy.

## Introduction

Normal trichromatic colour vision is colour constant, i.e., has the ability to partially discount the effects of the colour of the illumination on the perception of the surface colours of objects. The phenomenon of colour constancy has been extensively studied in normal colour vision [1] but has been little studied in defective colour vision, in particular in dichromacy, where the absence of one cone photopigment class severely impairs colour discrimination [2].

The most common form of dichromacy is red-green (R-G) dichromacy which is a genetically determined abnormality of the retinal pigments sensitive to medium (M pigment) or long (L pigment) wavelengths, affecting around 2% of males and 0.02% of females [2]. R-G dichromats are classified as protanopes or deuteranopes. Protanopia is caused by the substitution of L-photopigment by M-photopigment in the L-cones; deuteranopia is caused by the substitution of M-photopigment by L-photopigment in the M-cones [3], and in some rare cases either may be caused by photoreceptor loss [4]. Consequently, for practical purposes R-G dichromats only have two types of functional cones: in addition to S-cones, protanopes have operative M-cones whereas deuteranopes have operative L-cones. Given the absence of one of the pigments, colour discrimination in R-G dichromats is impaired along the R-G confusion lines [5] resulting in the perception of only a small gamut of the colours normal observers can perceive [6,7]. The famous description by John Dalton (a deuteranope himself [8]) of the pink flowers of a cranesbill, which appeared to him sky-blue by daylight but near yellow by candlelight [9], suggests that colour constancy may also be impaired in dichromacy.

In normal colour vision colour constancy has been experimentally accessed in diverse ways (for a detailed review, see Foster [1]): by asymmetric colour matching, colour naming, achromatic adjustment and by the performance in tasks requiring discrimination of illuminant changes from surface-reflectance changes. In dichromatic colour vision, however, studies have mainly used discrimination of illuminant changes from surface-reflectance changes and both achromatic matches and asymmetric paper matches. In the former, R-G dichromats showed less constancy than normal controls with Munsell samples [10,11] but almost normal constancy with natural scenes [11]. In experiments using achromatic matching, similar colour constancy was found for normal observers and R-G dichromats in the three axes tested [R-G cardinal axis, yellow-blue (Y-B) cardinal axis and Planckian locus] despite the R-G dichromats’ poorer colour discrimination in the R-G axis [12]. In experiments based on paper matches, a similar degree of adaptation to the illuminant was found for normal and R-G dichromats [13]. Together, this research suggests that colour constancy is, at least for natural surfaces and natural illuminants (and contrary to what could be inferred from John Dalton’s description of the colour of cranesbills), as efficient in dichromacy as in normal trichromacy. None of these studies, however, tested colour constancy with real natural images and global illuminant changes.

The aim of the work reported here was to test colour constancy in dichromacy with natural images and natural illuminants. Spectral reflectance data from natural images were obtained by hyperspectral imaging and used to build the stimuli. The experiments were based on an experimental paradigm reported recently [14,15] which estimates thresholds to perceive changes in a scene due to global illuminant changes. This experimental methodology is particularly well suited to test colour constancy on natural viewing conditions as it measures the sensitivity to detect chromatic changes in a complex natural image.

## Methods

### Stimuli

**Fig 1:**
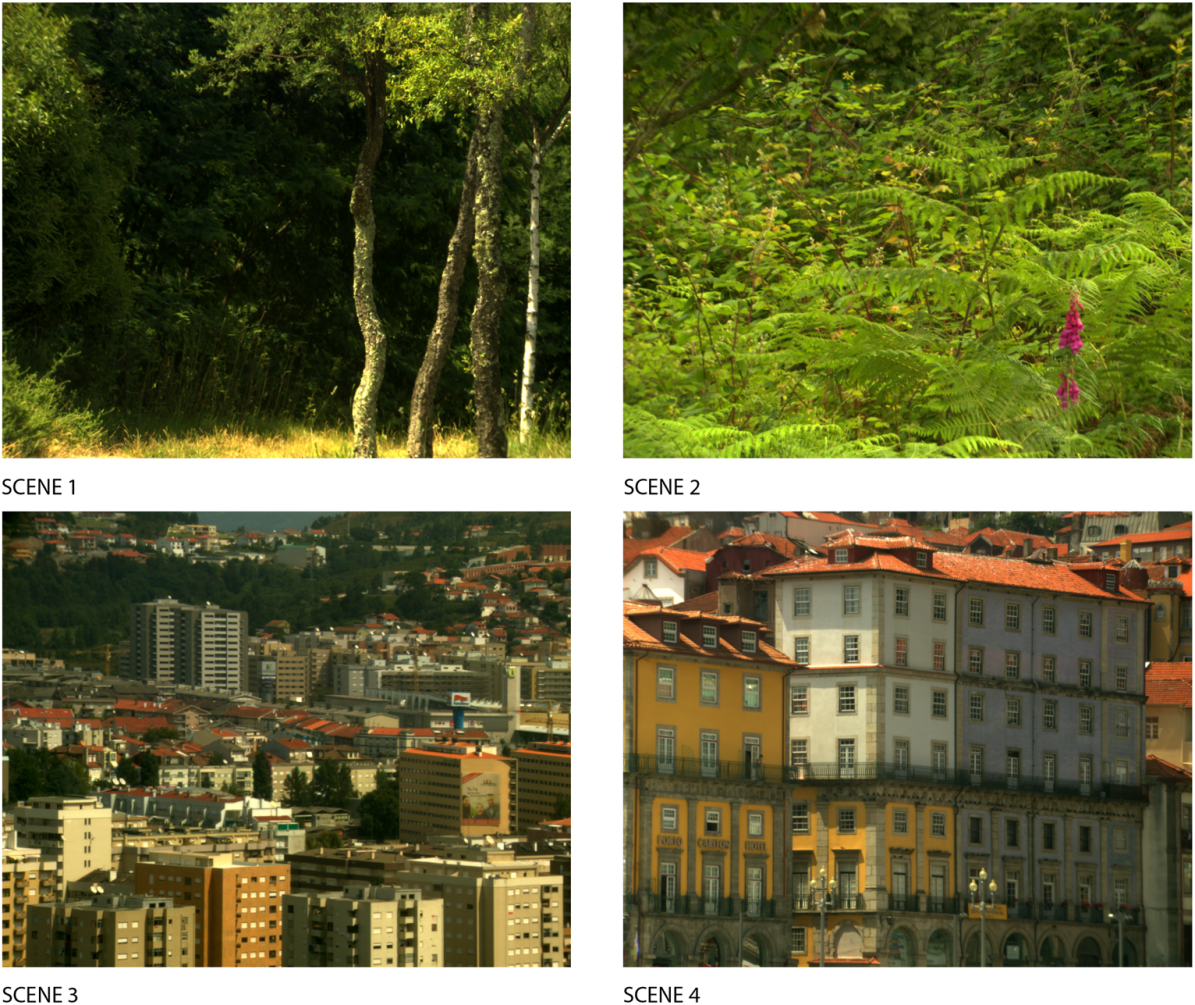
Images of the scenes used as stimuli rendered under the standard illuminant D67. The images were retrieved from a database of images with hyperspectral data of natural scenes [16] which can be freely accessed and used from: http://online.uminho.pt/pessoas/smcn/hsi_2004/hsi_2004.html.

Images of the scenes used in this study were rendered using hyperspectral imaging of natural scenes. The spectral range used was from 400 to 720 nm, with a spectral resolution of 10 nm. Two scenes of rural and two scenes of urban environments (as depicted in Fig 1) were selected from an existing database [16]. The images were presented on a CRT colour display, subtended an angle of 10° in the horizontal axis and were observed at 60 cm. Images were trimmed about 70 pixels from their original resolution to delete the reflectance standard reference from the scene and to match them in resolution. Images were also subsampled every other pixel from the original resolution of 1270x1017 pixels, so the image rendering system was able to cope with the images’ digital size. To simulate the effect of different illuminants on the images, the reflectance spectrum of each pixel of the scenes was multiplied by each illuminant spectrum. Illuminants were simulations of daylight of variable correlated colour temperature (CCT) synthetized from Judd’s daylight spectral basis functions [17].

**Fig 2:**
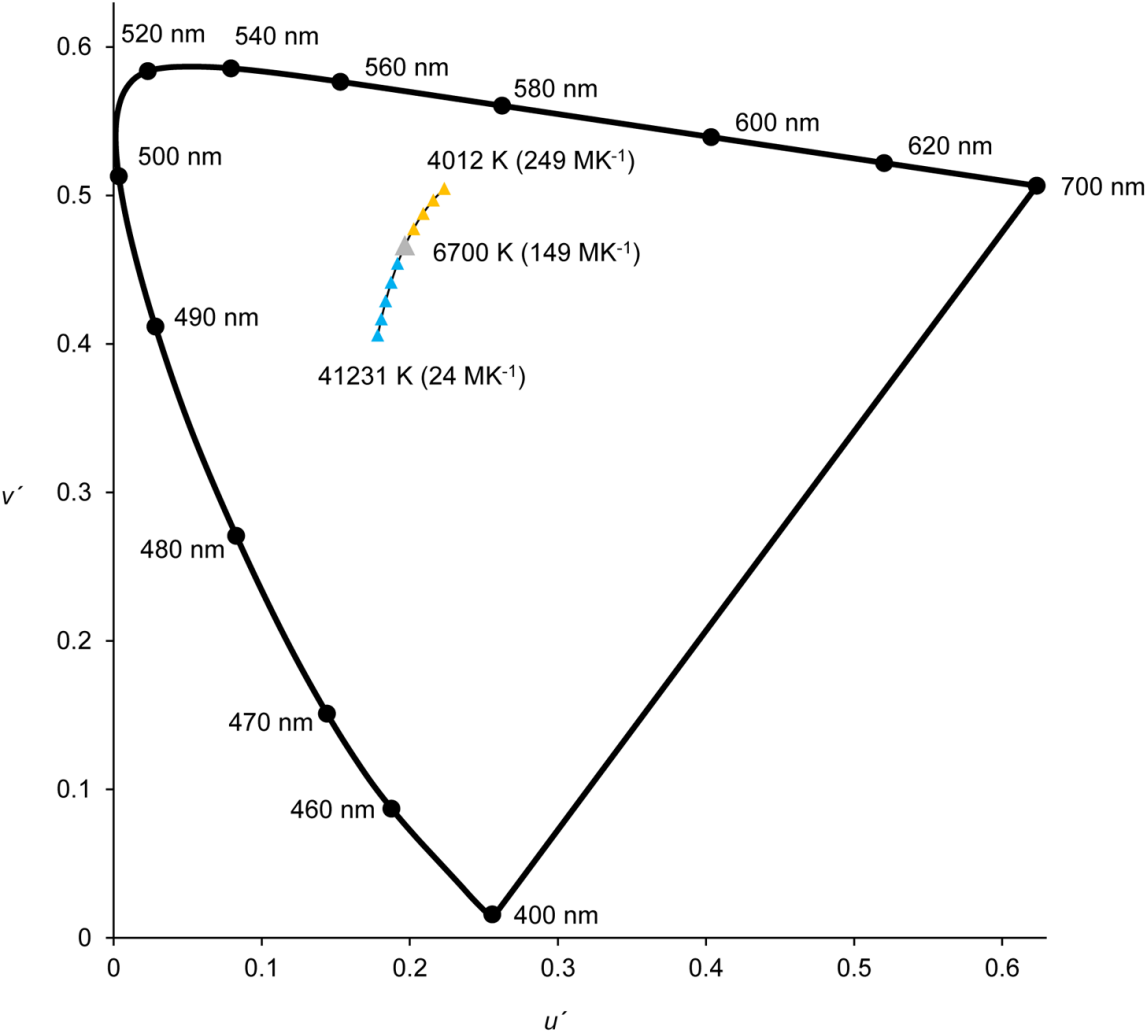
Daylight illuminants used in condition 1. In this condition only the colour of the illumination changed. The reference illuminant was always D67 (grey triangle). Each scene was rendered with a CCT in the range 4012–41231 K (249.25–24.25 MK^-1^; yellowish illuminants are represented by yellow triangles; bluish illuminants are represented by blue triangles), in steps of 25 MK^-1^.

There were two experimental conditions applied. In condition 1 the colour of the simulated illumination was changed. Each scene was rendered assuming an illuminant with a CCT in the range 4012–41231 K (249.25–24.25 MK^-1^) in 10 steps, equally spaced by 25 MK^-1^ (see Fig 2; where yellowish illuminants are represented by yellow triangles and bluish illuminants are represented by blue triangles). The CCT was spaced in reciprocal colour temperature (MK^-1^) to ensure visual uniformity [5]. The average luminance was kept constant at 10 cd/m^2^. In condition 2 the intensity of the simulated illumination was changed. Each scene was rendered with a constant CCT of 6700 K (149.25 MK^-1^, selected as the neutral point of the CCT testing interval) and the average luminance was adjusted in the range of 6–15 cd/m^2^ in 10 steps equally spaced by 1 cd/m^2^. For all conditions and scenes tested at least ≈90% of the pixels above 10 cd/m^2^ were inside the screen colour gamut or the displayed colour was deviated less than 2 JND (ΔE*_ab_ ≤ 2.3) from the intended colour. The ΔE*_ab_ = 2.3 value was around the threshold of chromatic discrimination for complex coloured images [18,19].

### Apparatus

The images of the scenes were displayed on a Sony–GDM F520 (Sony Corporation, Tokyo, Japan) controlled by a video card (ViSaGe Visual Stimulus Generator; Cambridge Research Systems, Rochester, Kent, UK) in 24-bits-per-pixel true-colour mode. The screen was calibrated in colour and luminance with a telespectroradiometer (PR-650 SpectraScan Colorimeter; Photo Research, Chatsworth, CA). A CB6 response box (Cambridge Research Systems, Rochester, Kent, UK) was used to register the observer’s responses.

### General procedure

The procedure was similar to that used by Pearce et al. [14] and Radonjić et al. [15] in a study with normal observers and real illuminants. The experiments were performed inside a dark room. At the beginning of each session, the observers viewed a uniform grey background with a CCT of 6700 K and a luminance of 10 cd/m^2^ for 5 minutes.

### Experiment 1: Thresholds for detecting a change

**Fig 3:**
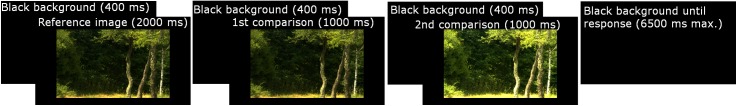
Stimuli sequence of an example trial. The reference image was always simulated rendered by D67. In condition 1 the CCT of the illumination changed in one of the intervals; in condition 2 the intensity of the illumination changed in one of the intervals. The observer’s task was to identify which of the intervals was different from the reference. Here a luminance change in the second image presentation is illustrated.

First experiment (a two-alternative forced-choice task) measured thresholds for detecting a change in the colour or in the intensity of the illuminant. Each observer performed 800 trials in one session: 4 scenes x 10 levels in each condition x 2 conditions (CCT or luminance change) x 10 repetitions. There were three automatic breaks every 200 trials (≈ 20 minutes). During the breaks the grey adaptation background was presented. All the trials had the same structure (see Fig 3). After a short presentation of a black background (400 ms), the reference image (6700 K and 10 cd/m^2^) appeared for 2 s. This was followed by another short presentation of the background (400 ms) and then the first comparison scene for 1 s. After another short presentation of the background (400 ms), the second comparison scene was presented for 1 s. Finally, a black background was presented until a response was made (6.5 s maximum). One of the comparison scenes was identical to the reference scene, i.e., with the same illumination; in the other either the CCT or the average luminance was different (see average luminance change of the second comparison image in Fig 3). The observers had simply to identify which of the two comparison scenes looked different from the reference scene.

### Experiment 2: Identification of the type of perceived changes

Second experiment (a two-alternative forced-choice task) identified how the changes are perceived by different types of observers. Each observer performed 160 trials: 4 scenes x 2 conditions (CCT or luminance change) x 2 supra-threshold levels in each condition (change towards higher or lower CCT/luminance) x 10 repetitions. All the trials had the same structure. After a short presentation of a black background (400 ms), the reference image (6700 K and 10 cd/m^2^) appeared for 2 s. It was followed by another short presentation of background (400 ms) and a comparison scene for 1 s. Finally, a black background was presented until a response was made (6.5 s maximum). The average illumination of the comparison scene was different from the illumination in the reference scene, but it presented either a pure CCT change or a pure luminance change. The extent of the CCT/luminance change was adapted for each observer/scene individually, using the threshold results from experiment 1. It was a supra-threshold change two steps above the 75% threshold computed from experiment 1, assuming the 10 levels in both conditions as described before. The observers were required to specify whether or not the change was only in intensity. Observers verbally informed the experimenter that they understood the task.

#### Discrimination thresholds task

A discrimination task [20] was used to estimate colour discrimination thresholds loci using a staircase procedure. Each observer was tested with 500 trials: 25 trials x 20 hues. Each trial presented a square chromatic target on an achromatic static luminance noise background. Observers were required to indicate the location of the square (right or left). Discrimination thresholds for each hue were measured on three occasions and averaged for each observer.

### Design

#### Experiment 1: Thresholds for detecting a change

A two-alternative forced-choice task measured discrimination thresholds (the dependent variable) in a 3x2x2x4 mixed-measures design. The independent variable group of observers (normal trichromats, protanopes and deuteranopes) was the between-subjects factor and the independent variables type of illumination change (CCT or luminance), change direction (higher or lower) and scene (four scenes, see Fig 1) were within-subject factors.

#### Experiment 2: Identification of the type of perceived changes

A two-alternative forced-choice task measured hit rate (the dependent variable) in the same 3x2x2x4 mixed-measures design as described for experiment 1.

### Observers

Eleven observers (4 colour normal, age range 25–36 y; 3 protanopes, age range 22–51 y; 4 deuteranopes, age range 22–47 y) participated in the experiments. A one-way ANOVA analysis showed no significant group differences in age (mean age: colour normal = 30.00 y, SD = 4.69; protanopes = 32.33 y, SD = 16.20; deuteranopes = 35.00 y, SD = 10.86), *F*(2,8) = 0.21, *p =* 0.81. All but two of the authors (LA and JML) were naïve to the experiments’ purposes. Each observer had normal or corrected-to-normal acuity and their colour vision was tested with Ishihara plates [21], the Farnsworth-Munsell 100 hue colour vision test [22], the Cambridge Colour Test [23], the Color Assessment & Diagnosis (CAD) test [24], an in-house adaptation [20] of the Universal Colour Discrimination Test (UCDT [25]) and performed Rayleigh match in an Oculus HMC anomaloscope ([26], see S1 Table for detailed results on colour vision tests). Observers were reimbursed for their participation in the study. The experiment was conducted in accordance with the Declaration of Helsinki and was granted ethical approval by the Ethics Committee of the University of Minho (Process SECVS 029/2014). Observers were asked to give written consent before participating in the study, and were informed of their right to withdraw at any time without penalty.

## Results

### Experiment 1

**Fig 4:**
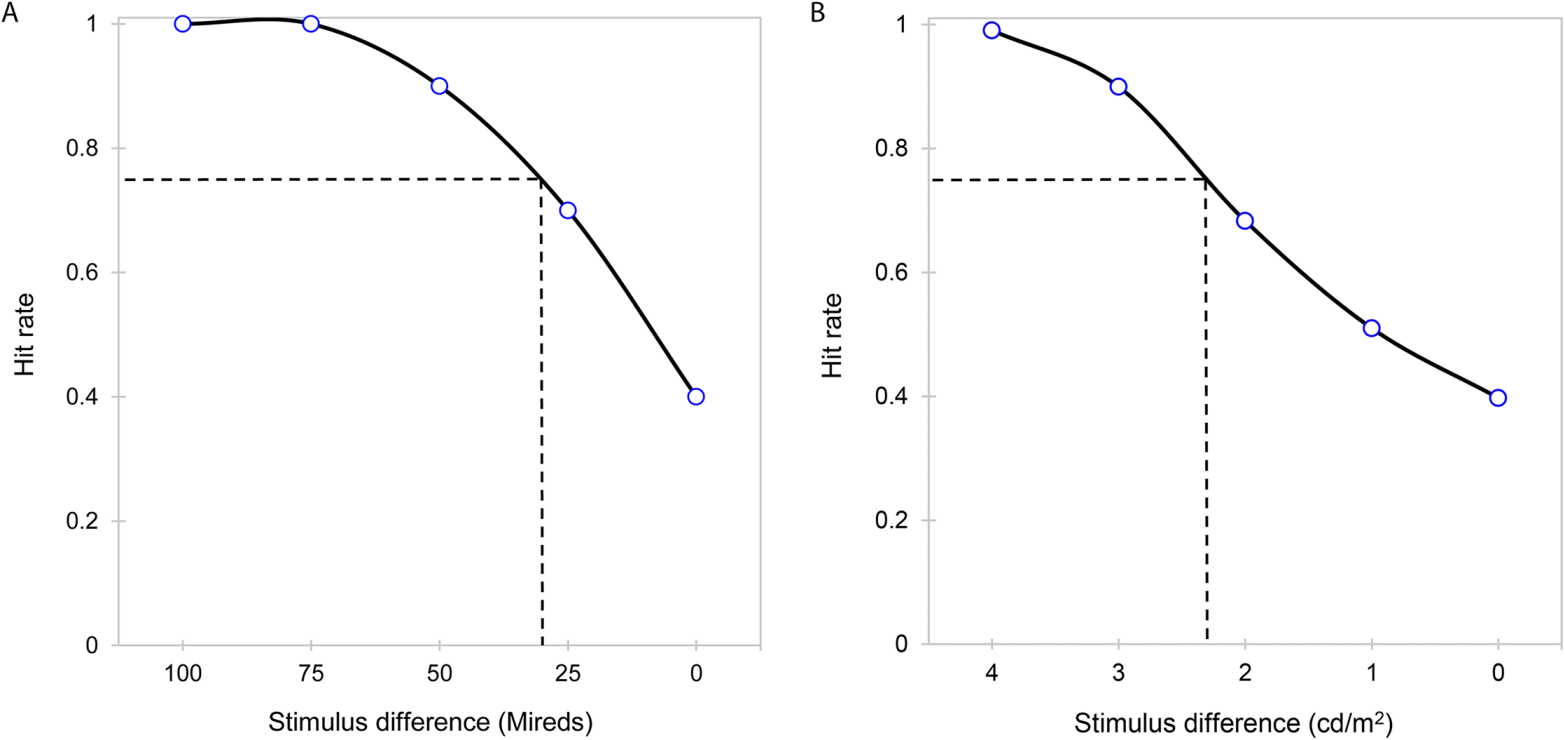
Examples of psychometric functions. Thresholds were computed based on a 75% criterion (dashed line) and extracted from fitted psychometric functions (solid line). Provided examples represent data from a protanope observer for a change towards higher CCT for scene 4 (Fig 4A) and a normal observer for a change towards lower luminance for scene 2 (Fig 4B).

A psychometric function was fitted to the data to extract thresholds using a criterion of 75% towards higher or lower CCT or luminance (see Fig 4). The function was fitted on a non-parametric approach which made no assumption about the shape of the true function underlying the experimental data except its smoothness [27].

#### Thresholds detecting changes on CCT

**Fig 5:**
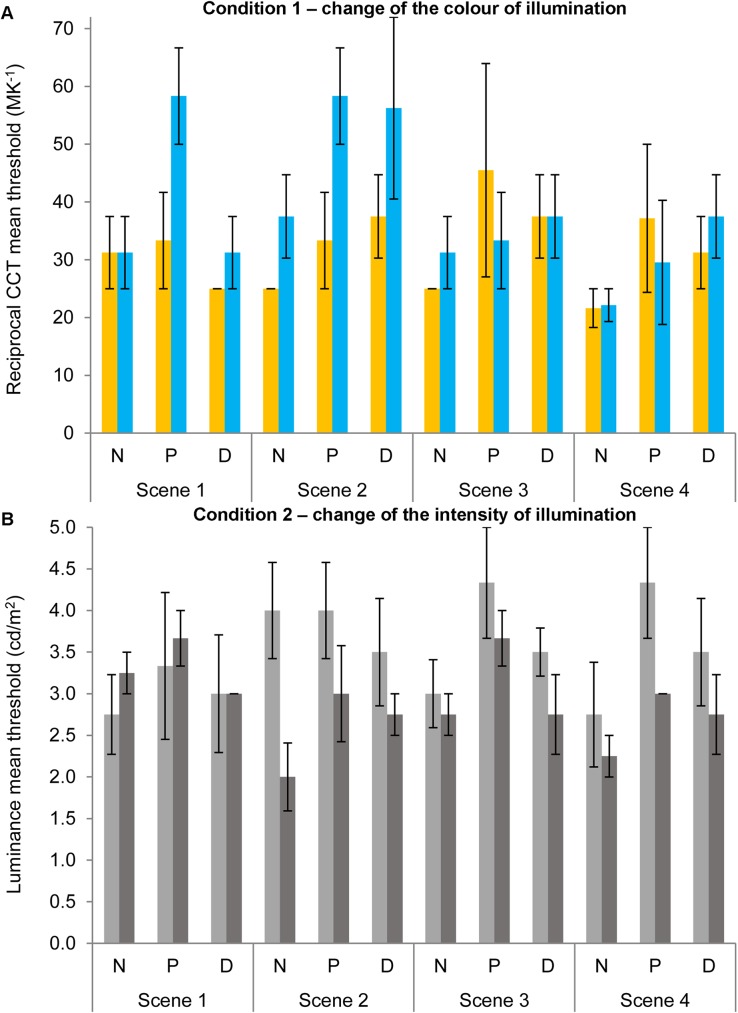
Thresholds for the four scenes in experiment 1. Mean reciprocal CCT (in MK^-1^, Fig 5A) or luminance (in cd/m^2^, Fig 5B) thresholds towards higher (yellow bars in 5A; light grey bars in 5B) or lower (blue bars in 5A; dark grey bars in 5B) values in relation to the reference scene (149.25 MK^-1^ and 10 cd/m^2^) for normal observers (N), protanopes (P) or deuteranopes (D) for the four scenes (see x-axis). Error bars show standard error of the mean (SEM). The lack of an error bar in some conditions/groups is due to lack of variability in the data.

**Fig 6:**
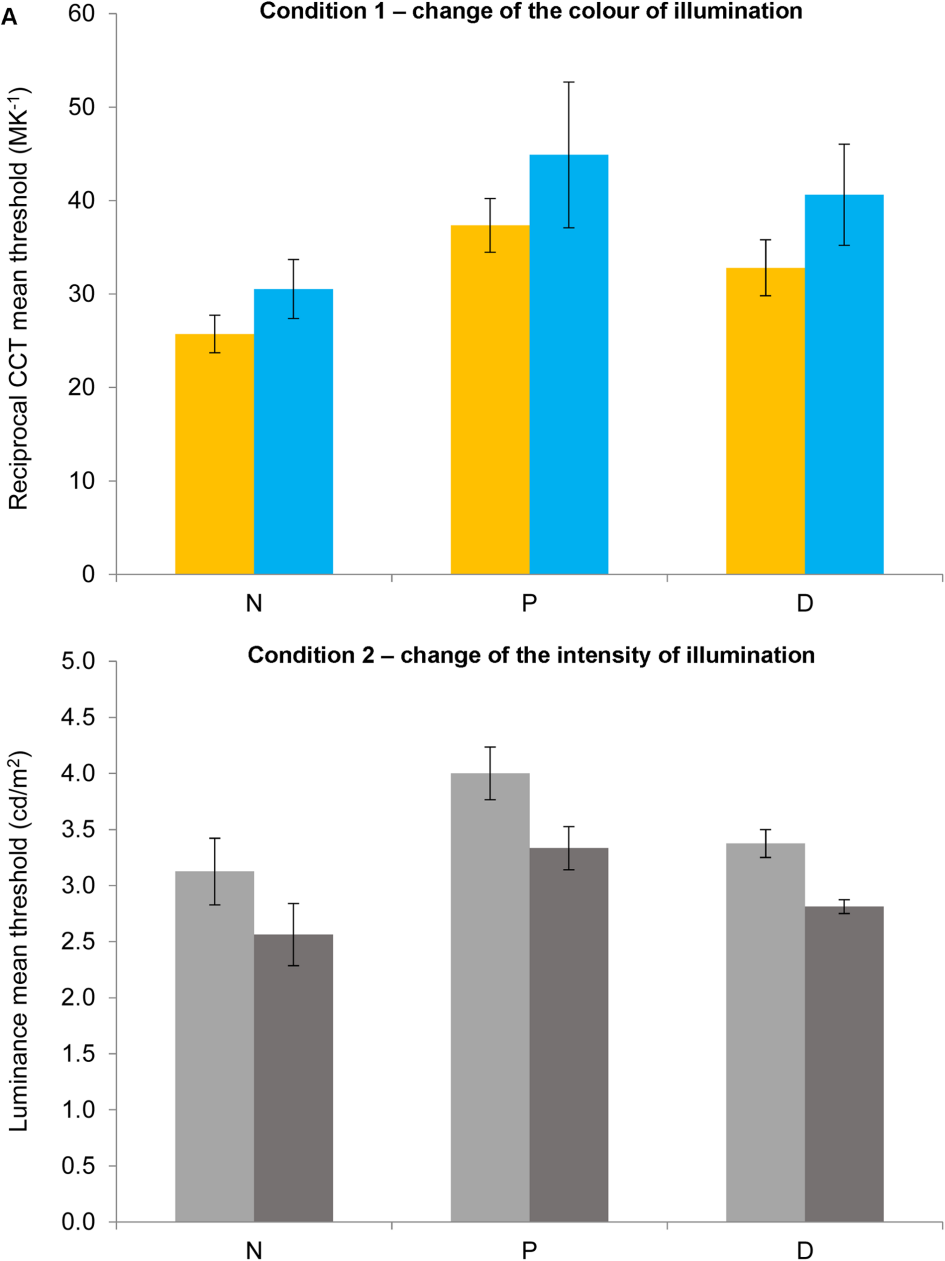
Thresholds for experiment 1 averaged across scenes. Mean reciprocal CCT (in MK^-1^, Fig 6A) or luminance (in cd/m^2^, Fig 6B) thresholds towards higher (yellow bars in 6A; light grey bars in 6B) or lower (blue bars in 6A; dark grey bars in 6B) values in relation with the reference scene (149.25 MK^-1^ and 10 cd/m^2^) for normal observers (N), protanopes (P) or deuteranopes (D) averaged across scenes. Error bars show SEM.

Fig 5 shows the mean thresholds for the two conditions of experiment 1 for changes towards higher (yellow or light grey bars) or lower (blue or dark grey bars) CCT or luminance for the four scenes for normal observers (N), protanopes (P) or deuteranopes (D). Bar colours exhibit the type of illuminant change represented by the thresholds: yellow bars for changes towards yellowish illuminants (lower CCT); blue bars for changes towards bluish illuminants (higher CCT); light grey bars for changes towards brighter illuminants; dark grey bars for changes towards dimmer illuminants. For simplicity, Fig 6 shows similar data to Fig 5 but which are averaged across scenes and observers for normal observers (N), protanopes (P) or deuteranopes (D).

A mixed-model ANOVA analysis was conducted with group (normal observers, protanopes and deuteranopes) as the between-subjects factor, and change direction (higher or lower) and scene (scene 1, 2, 3 4) as within-subject factors on the estimated thresholds for CCT changes in relation to the reference scene (149.25 MK^-1^, see Fig 5A; Normality of variables was confirmed by Kolmogorov-Smirnov tests, all *p>*.05). This analysis did not show any significant effect of change direction, scene, group or their interactions (all *p>*.05). In order to increase statistical power, the same analyses were repeated but combining both dichromat groups (between-subjects factor with two levels: normal and dichromat). This analysis did reveal a significant effect of group [two levels, *F*(1,9) = 6.90, *p <* .05, *η*^*2*^ = .43, higher thresholds for dichromats (38.60 MK^-1^) in relation to normal observers (28.13 MK^-1^)] but not significant effects of change direction, scene, or their interactions (all *p>*.05).

#### Thresholds detecting changes in intensity

A mixed-model ANOVA analysis was conducted with group (normal observers, protanopes and deuteranopes) as the between-subjects factor, and change direction (higher or lower) and scene (scene 1, 2, 3 4) as within-subject factors on the estimated thresholds for luminance changes in relation to the reference scene (10 cd/m^2^, see Fig 5B). This analysis did not show any significant effect of change direction, scene, group or their interactions (all *p>*.05) except for an effect of the interaction of change direction and scene [*F*(3,24) = 4.13, *p <* .05, *η*^*2*^ = .34]. Bonferroni-corrected pairwise comparisons showed significant differences only on scene 2 towards higher thresholds for the lighter (3.82 cd/m^2^) than for the darker (2.55 cd/m^2^) direction. In order to increase statistical power, the same analyses were repeated but combining both dichromat groups (between-subjects factor with two levels: normal and dichromat) but there were no significant effect of change direction, scene, group or their interactions (all *p>*.05) except for the same effect of the interaction of change direction and scene [*F*(3,27) = 5.28, *p <* .04, *η*^*2*^ = .37] (Bonferroni-corrected pairwise comparisons showed significant differences only on scene 2 towards higher thresholds for the lighter direction).

#### Comparison between colour and illuminant discrimination thresholds

**Fig 7:**
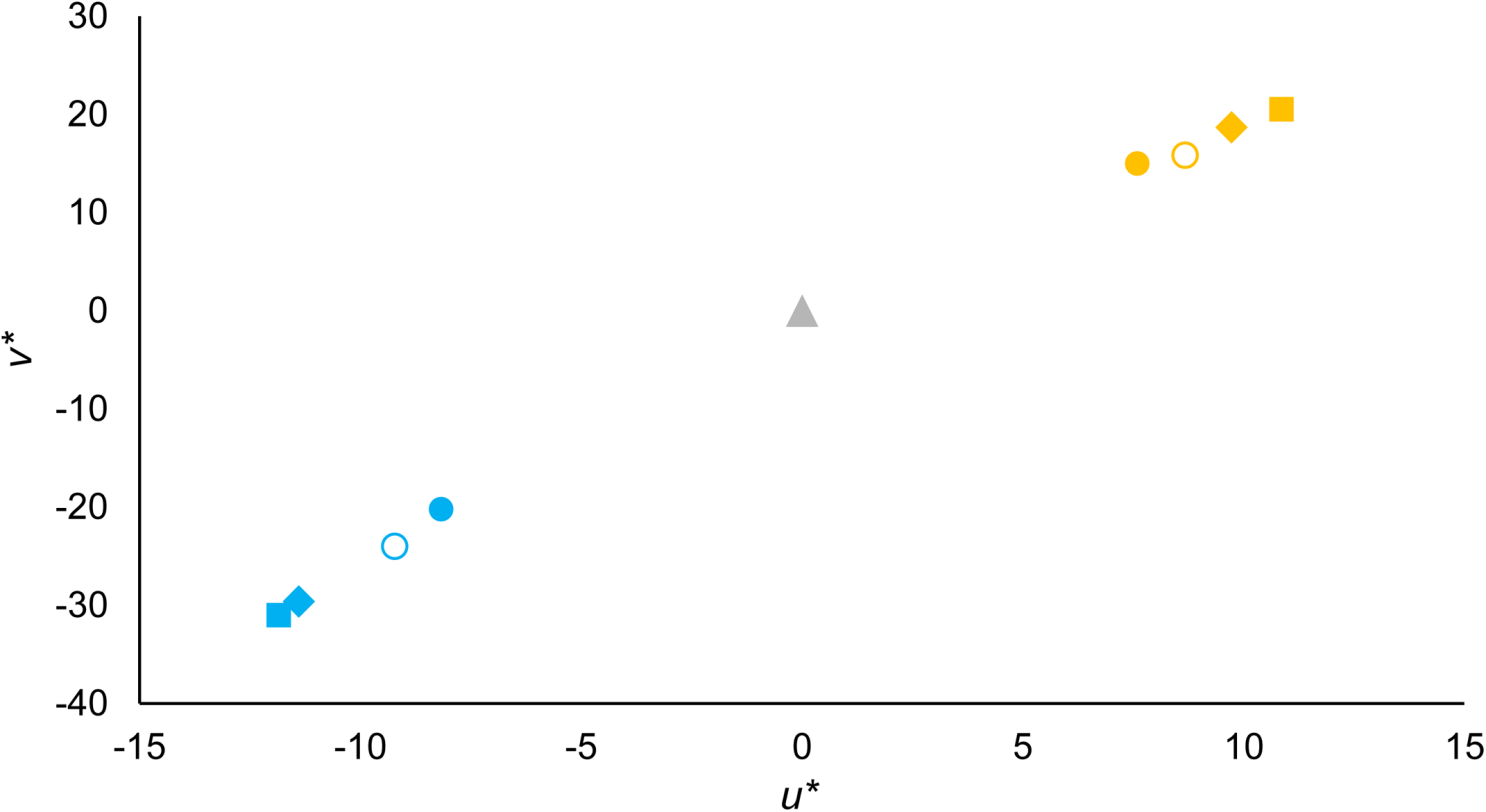
Mean thresholds for illuminants along daylight locus, plotted in CIE u*v* coordinates. Reference white corresponds to D67 illuminant (u* = 0, v* = 0, grey triangle). Data for normal observers in Pearce et al. ([14], open circles) in comparison with normal observers (solid circles), protanopes (squares) and deuteranopes (diamonds) in this study. Yellowish illuminants are represented as yellow symbols; bluish illuminants are represented as blue symbols.

Fig 7 compares CCT discrimination thresholds from the current experiment with those from Pearce et al. (see Fig 2C in [14]) in CIELUV colour space (u* and v* units were used to allow a direct comparison). The results are similar for normal observers in both experiments and also between different types of observers in the current experiment, especially for yellowish illuminants. The small differences may result from different sampling of the background colours [14,15].

**Fig 8:**
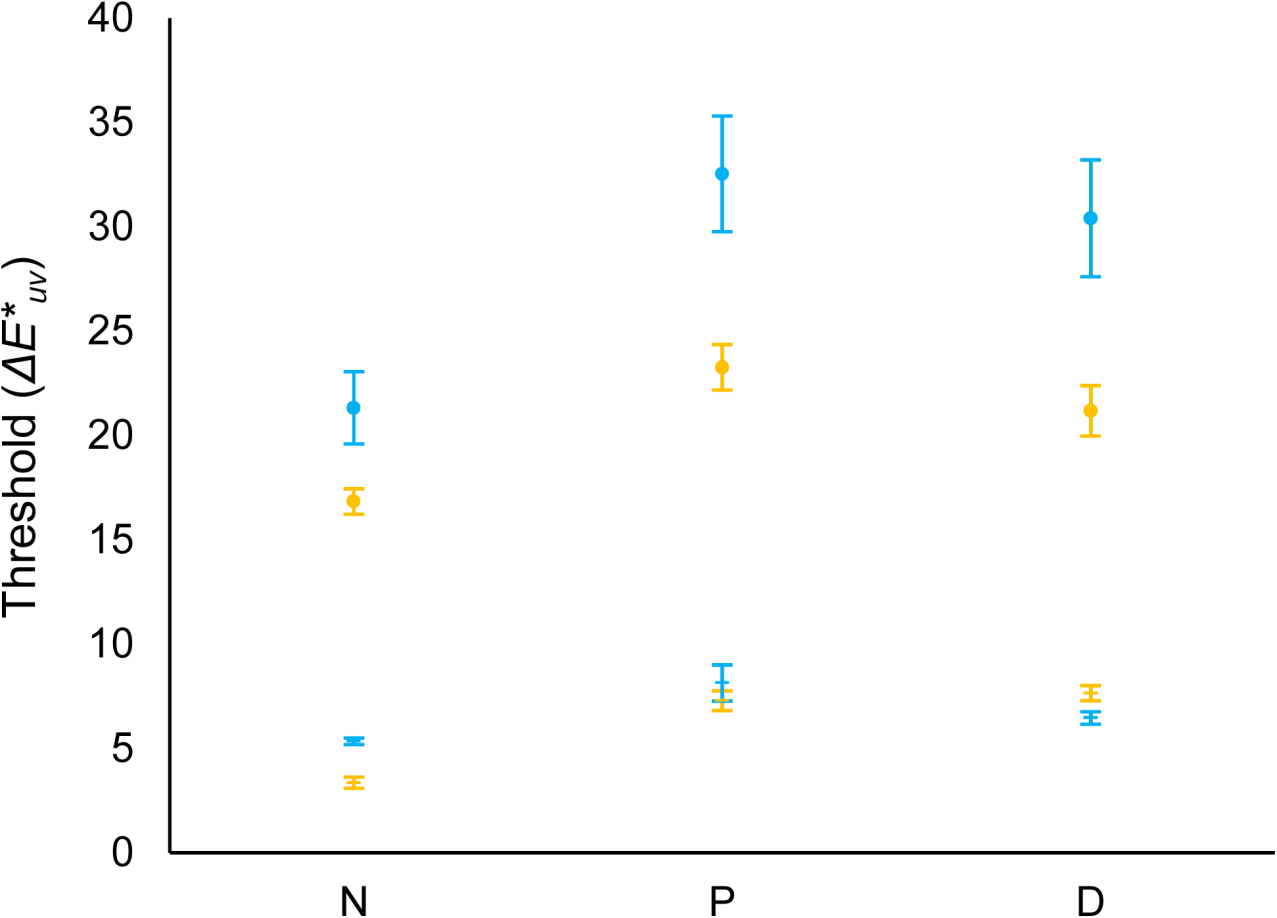
Mean illuminant discrimination and mean colour discrimination thresholds along daylight locus. Circles correspond to thresholds in ΔE*_uv_ for illuminants along daylight locus for the two illumination change directions of the CCT condition (towards yellowish illuminants, yellow circles; towards bluish illuminants, blue circles) of experiment 1 in relation with the reference illuminant (*u´*, = 0.1968; *v´* = 0.4663; *Y* = 10 cd/m^2^). Dashes correspond to the colour discrimination thresholds along daylight locus in ΔE*_uv_ for the two illumination directions in Fig 2 (yellowish illuminants, yellow dashes; bluish illuminants, blue dashes) in relation with the reference white (*u´*, = 0.1947; *v´* = 0.4639; *Y* = 11 cd/m^2^). Mean thresholds correspond to normal observers (N), protanopes (P) or deuteranopes (D, see x-axis). Error bars show SEM.

Fig 8 shows the mean thresholds transformed to ΔE*_uv_ units for the CCT condition (values from Fig 6A) of experiment 1 for the two illumination change directions (towards yellowish illuminants, yellow circles; towards bluish illuminants, blue circles) in relation with the reference illuminant (*u´*, = 0.1968; *v´* = 0.4663) for normal observers (N), protanopes (P) or deuteranopes (D). Fig 8 also represents the colour discrimination thresholds along daylight locus (yellow/blue dashes). These discrimination thresholds loci were estimated from the discrimination ellipses obtained using an in-house adaptation [20] of the UCDT [25]. The two intersections (bluish/yellowish illuminants) between the individual’s discrimination ellipse and the corresponding illuminant segment of the line represented in Fig 2 were calculated. These values are the estimations of the colour discrimination thresholds along daylight locus in Fig 8 (blue/yellow dashes).

A mixed-model ANOVA analysis was conducted with group (normal observers, protanopes and deuteranopes) as the between-subjects factor and discrimination type (illuminant or chromatic discrimination, circles and dashes in Fig 8) and direction (yellowish or bluish) as within-subject factors on the estimated ΔE*_uv_ thresholds. This analysis revealed a significant main effect of discrimination type [*F*(1,8) = 201.67, *p <* .001, *η*^*2*^ = .96; lower thresholds for chromatic discrimination (ΔE*_uv_ = 6.38) in relation to illuminant discrimination (ΔE*_uv_ = 24.26)], of direction [*F*(1,8) = 8.40, *p <* .05, *η*^*2*^ = .51; higher discrimination thresholds towards bluish direction (ΔE*_uv_ = 17.37) in relation to yellowish direction (ΔE*_uv_ = 13.26)] and of group of observers [*F*(2,8) = 4.52, *p <* .05, *η*^*2*^ = .53; Bonferroni-corrected pairwise comparisons did not show significant differences between the three groups; LSD pairwise comparisons showed higher thresholds for protanopes (ΔE*_uv_ = 17.81) and deuteranopes (ΔE*_uv_ = 16.43) in relation to normal observers (ΔE*_uv_ = 11.72), both *p <* .05] but no effect of their interactions (all *p>*.05). There was no significant Pearson correlation between the yellow or bluish illuminant discrimination thresholds and the colour discrimination thresholds for none of the groups of observers either collapsing or not collapsing the two dichromat groups (*R*^*2*^ = 0.01–0.41, all *p>*.05, see S1 Fig).

### Experiment 2

In order to discard that dichromat observers could use their particular intensity perception as a cue to detect the CCT changes, experiment 2 required the observers to identify if the change was a pure luminance change or not.

**Fig 9:**
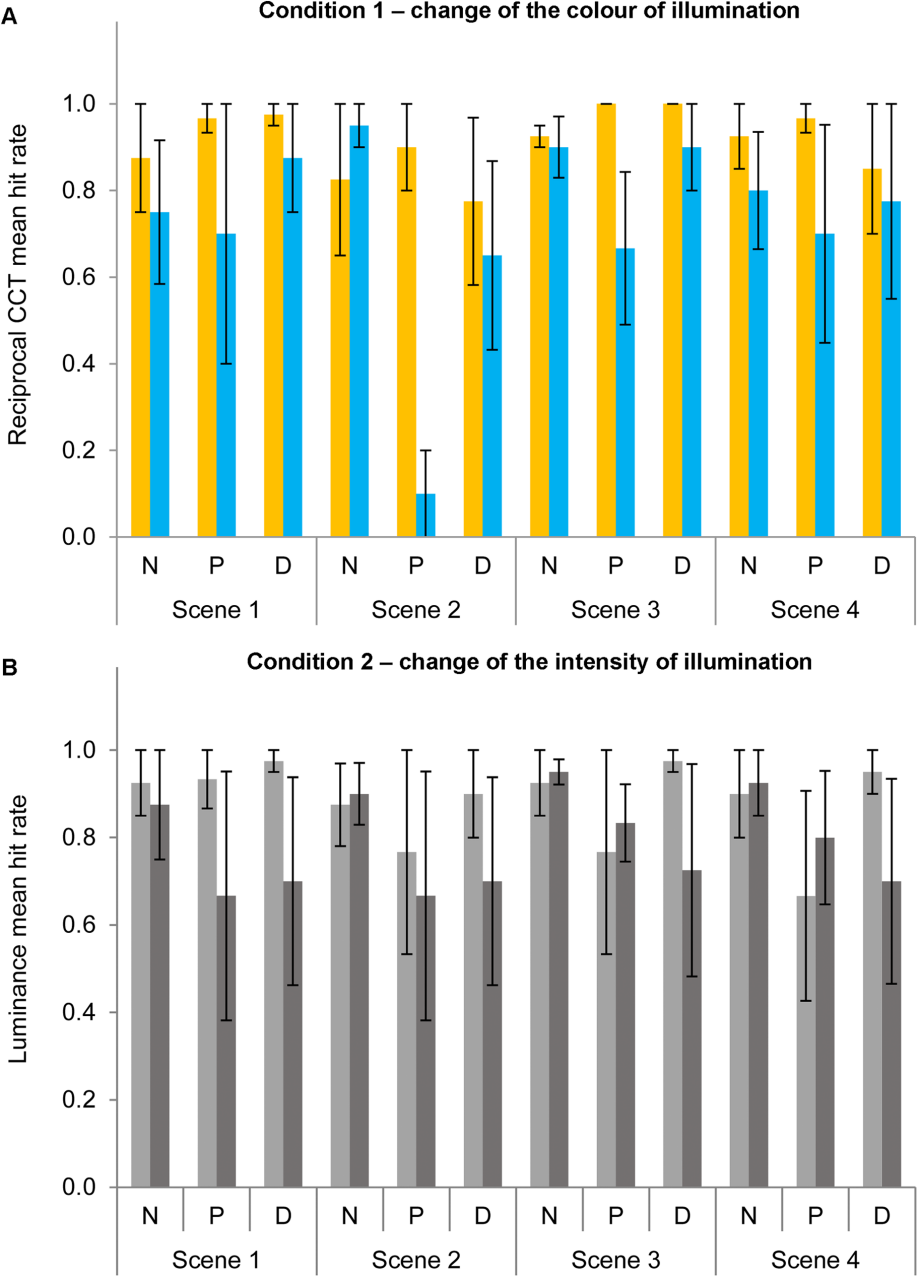
Mean hit rates for the four scenes in experiment 2. Mean reciprocal CCT (Fig 9A) or luminance (Fig 9B) change identification hit rate towards higher (yellow bars in 9A; light grey bars in 9B) or lower (blue bars in 9A; dark grey bars in 9B) values for normal observers (N), protanopes (P) or deuteranopes (D) for the four scenes (see x-axis). Error bars show SEM. The lack of an error bar in some conditions/groups is due to lack of variability in the data.

**Fig 10:**
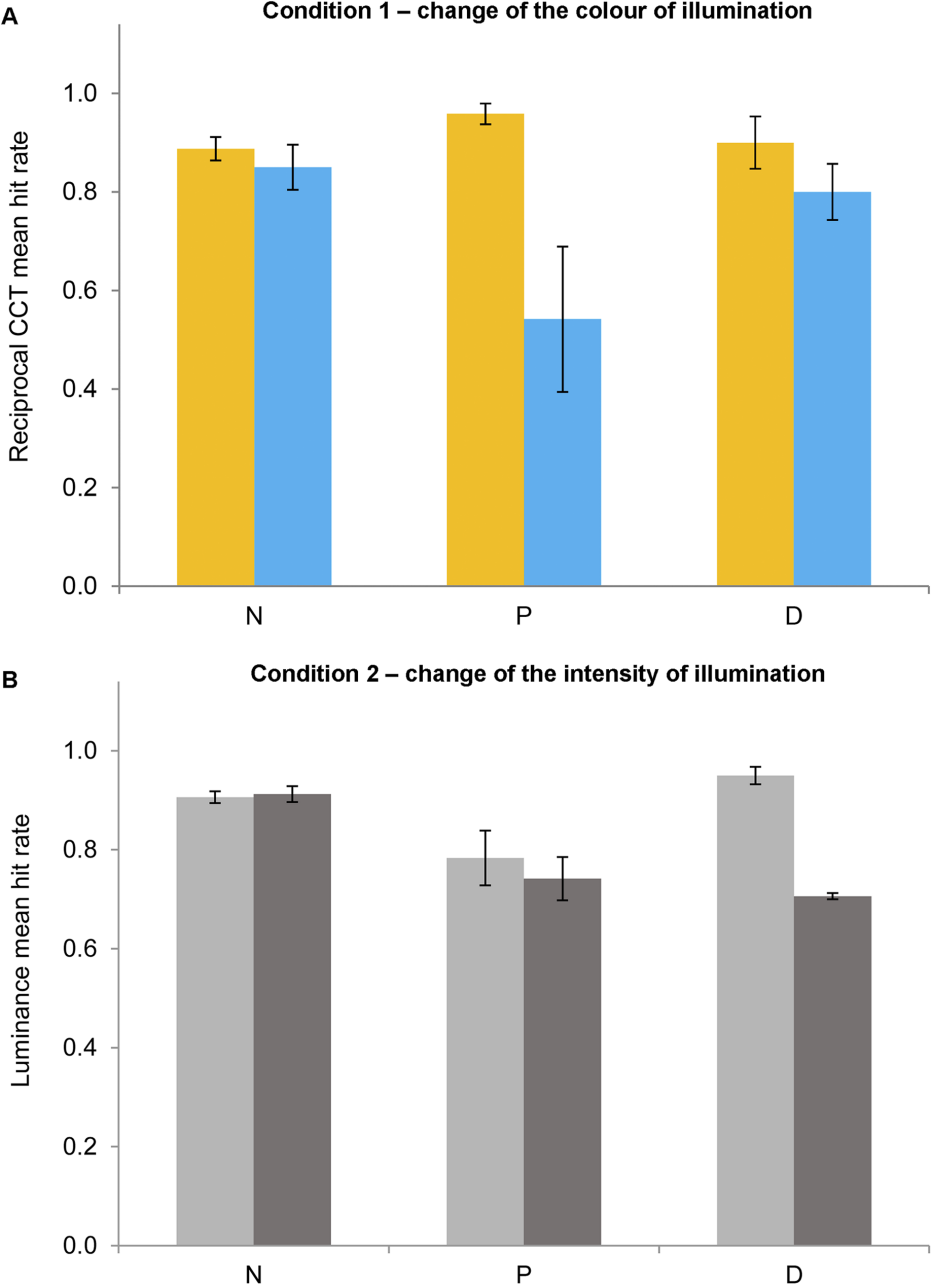
Mean hit rates for experiment 2 averaged across scenes. Mean reciprocal CCT (Fig 10A) or luminance (Fig 10B) change identification hit rate towards higher (yellow bars in 10A; light grey bars in 10B) or lower (blue bars in 10A; dark grey bars in 10B) values for normal observers (N), protanopes (P) or deuteranopes (D) averaged across scenes. Error bars show SEM.

Fig 9 shows the mean hit rates for the two conditions of illuminant change identification of experiment 2 (reciprocal CCT, Fig 9A; luminance, Fig 9B) towards higher (yellow or light grey bars) or lower (blue or dark grey bars) CCT or luminance in relation to the reference scene (149.25 MK^-1^ and 10 cd/m^2^) for the four scenes for normal observers (N), protanopes (P) or deuteranopes (D). For simplicity, Fig 10 shows similar data to Fig 9 but averaged across scenes and observers for normal observers (N), protanopes (P) or deuteranopes (D).

A mixed-model ANOVA analysis was conducted with group (normal observers, protanopes and deuteranopes) as the between-subjects factor and type of change (CCT or luminance), change direction (higher or lower) and scene (scene 1, 2, 3 4) as within-subject factors on the hit rates obtained in experiment 2, with the Greenhouse–Geisser correction applied when appropriate. This analysis showed a significant effect of scene [*F*(3,24) = 3.52, *p <* .05, *η*^*2*^ = .31], and of the interaction between type of change and direction [*F*(1,8) = 8.32, *p <* .05, *η*^*2*^ = .51], but no effect of type of change, change direction, scene, group of observers or any other interaction (all *p>*.05). Bonferroni-corrected pairwise comparisons did not show significant differences between the four scenes used [LSD pairwise comparisons showed a significantly lower hit rate for scene 2 (.75) in relation to scenes 1 (.85) and 3 (.88), both *p <* .05] but showed differences between the two change directions only in the CCT condition [higher hit rate for changes towards yellowish illuminants (.92) in relation to bluish illuminants (.73)]. In order to increase statistical power, same analyses were repeated but combining both dichromat groups (between-subjects factor with two levels: normal and dichromat), with the Greenhouse–Geisser correction applied when appropriate. This analysis did not reveal any significant effect of change type, change direction, scene, group or their interactions (all *p>*.05).

## Discussion

The main purpose of this work was to test how normal trichromats and colour deficient observers perceived changes in the colour or the intensity of illuminants on complex images of natural scenes. It was found that R-G dichromats detect natural changes in the colour of the illumination in natural scenes slightly worse than normal trichromats do. The magnitude of the difference between normal trichromats and dichromats was 10.47 MK^-1^ which is far from the average threshold for normal observers towards bluish (30.54 MK^-1^) or yellowish direction (25.72 MK^-1^). Thus illuminant discrimination thresholds of R-G dichromats are comparable to those of normal observers. No differences were found regarding the detection of changes in the intensity of illuminants. Thus R-G dichromats detect natural changes in the intensity of the illumination in natural scenes at the same level as normal trichromats do. The CCT and luminance discrimination thresholds were similar independently of the scenes used, which implies that the type of scene does not impact the final outcome. These results suggest that colour constancy mechanisms are robust for R-G dichromats over the daylight illuminants tested and are comparable to those of normal trichromats, a result supported on the basis of the filtering properties of the dichromatic colour system [28]. Although these constancy levels hold for natural lighting, it is unclear whether they hold for other more artificial lighting such as those tested by Pearce et al. [14].

In the experimental paradigm adaptation may not be complete because the times involved in the presentation of the stimuli are short. Detection of changes could therefore be driven by single surface detection mechanisms and be unrelated to colour constancy. However, thresholds for detecting CCT changes were much larger than thresholds for colour discrimination in the same area of the colour space (both differed in 17.88 ΔE*_uv_ units). Moreover, there was no linear relation between both types of thresholds in any observers’ group. Thus, colour discrimination and illuminant discrimination are unlikely to be related in this task. There has been some debate about the strong colour constancy mechanisms in R-G dichromats arising from the weaker chromatic discrimination in these observers [12]. The dissimilarity of the CCT and the chromatic discrimination thresholds suggests the action of independent mechanisms for colour discrimination and colour constancy both in normal observers and in R-G dichromats.

In experiment 2 it is shown that R-G dichromats also identify the quality of illumination changes, colour or intensity, in the same way as normal trichromats. This rules out the possibility of intensity changes being used as a cue to identify colour changes in our experiments, as suggested in other context by several works as Jameson and Hurvich [29] or Wachtler, Dohrmann and Hertel [30].

The task used in the first experiment was similar to the task previously used by Pearce et al. [14] and Radonjić et al. [15] with normal trichromats. Fig 7 shows high similarity in the mean discrimination accuracy along the yellow-blue line between the normal observers of this study and those of Pearce et al. [14]. Although the task was the same in both studies, the stimuli were quite different. However, the similarity in the overall results suggests that both studies measured the same colour constancy mechanisms in normal observers, again despite the different stimuli.

Colour constancy mechanisms of normal observers both in the current study and in previous research [14,31,32] are optimised for bluish illuminants. This trend also appears for the R-G dichromats included in this study. However, our data do not support the influence of stimulus content in the CCT discrimination thresholds, as has been also previously reported [15]. The absence of a scene effect in the current research may arise from a lack of power due to a limited number of observers. This fact along with the impossibility of capturing a difference between normal trichromats and the two groups of dichromats as separate groups suggest that future research will benefit of a larger number of observers and scenes.

These results confirm previous research [10,11] proposing strong colour constancy mechanisms in R-G dichromats with natural scenes and illuminants along the daylight locus, and also other studies suggesting independency between chromatic discrimination and colour constancy [12,32].

## Supporting information

S1 Table Detailed results for colour vision tests. (DOCX)

S1 Fig Relation between illuminant and colour discrimination thresholds along daylight locus for each group of observers. Colour discrimination thresholds in ΔE*_uv_ along daylight locus (y-axis) for the two illumination directions in Fig 2 (towards yellowish illuminants, panels A-D; towards bluish illuminants, panels E-H) in relation with the reference white (*u´*, = 0.1947; *v´* = 0.4639; *Y* = 11 cd/m^2^), for normal observers (panels A and E, solid circles), protanopes (panels B and F, squares), deuteranopes (panels C and G, diamonds) and both dichromat groups collapsed (panels D and H, triangles) against illuminant discrimination thresholds in ΔE*_uv_ along daylight locus (x-axis) for the two illumination change directions of the CCT condition (towards yellowish illuminants, panels A-D; towards bluish illuminants, panels E-H) of experiment 1 in relation with the reference illuminant (*u´*, = 0.1968; *v´* = 0.4663; *Y* = 10 cd/m^2^). *R*^*2*^ (all *p>*.05) and the best-fitting lines derived from linear least-squares regressions are also given.
(TIF)

S1 Dataset Thresholds obtained in experiment 1 and on the discrimination task. (PDF)

S2 Dataset Hit rate obtained in experiment 2. (PDF)
