# Supplementary material for: Robust colour constancy in red-green dichromats
Source: PLoS One. 2017 Jun 29;12(6):e0180310. doi: 10.1371/journal.pone.0180310 (PMC5491172; doi:10.1371/journal.pone.0180310)
Supplement: S1 Table — (DOCX) [file pone.0180310.s001.docx]

**Table S1. Detailed results for colour vision tests.**

| ID^a^ | Ishihara^b^ | FM-100^c^ | CCT^d^ | CAD^e^ | HMC^f^ |
| --- | --- | --- | --- | --- | --- |
| N1 | Pass | 5 | 1.15 | 1.18/1.02 | 38.6-44.1/16.6-14.5 |
| N2 | Pass | 6 | 1.04 | 1.16/1.06 | 44.7-45.8/14.6-14.1 |
| N3 | Pass | 8 | 1.39 | 1.06/1.00 | 40.4-44.9/12.9-12.2 |
| N4 | Pass | 6 | 1.08 | 1.28/1.18 | 44.7-45.1/13.6-11.8 |
| P1 | Fail | 76 | 14.86 | 22.48/0.98 | 0-73/34.1-2.7 |
| P2 | Fail | 52 | 5.86 | 24.78/1.27 | 0-73/28.65-2.69 |
| P3 | Fail | 62 | 9.22 | 25.51/1.37 | 0-73 /22.51-2.51 |
| D1 | Fail | 86 | 16.81 | 25.37/2.07 | 0-73/12.67-16.66 |
| D2 | Fail | 54 | 6.65 | 14.62/1.02 | 0-73/16.52-16.37 |
| D3 | Fail | 98 | 471.00 | 24.47/1.17 | 0-73/13.37-12.74 |
| D4 | Fail | 60 | 725.42 | 22.16/1.93 | 0-73/9.98-14.66 |

^a^N, P and D stand for Normal, Protanope and Deuteranope.

^b^Pass/Fail result is provided for Ishihara.

^c^Total error score is provided for Farnsworth-Munsell 100 (FM-100).

^d^D axis ratio value is provided for Cambridge Colour Test (CCT).

^e^R-G/Y-B “normal” mean thresholds are provided for Color Assessment & Diagnosis (CAD). The “normal CAD threshold unit” describes the colour signal strength for the average normal trichromatic observer.

^f^Matching/luminance range is provided for the Rayleigh match in the Oculus HMC anomaloscope (HMC).
