# Supplementary figures and images for: Robust colour constancy in red-green dichromats
Source: PLoS One. 2017 Jun 29;12(6):e0180310. doi: 10.1371/journal.pone.0180310 (PMC5491172; doi:10.1371/journal.pone.0180310)

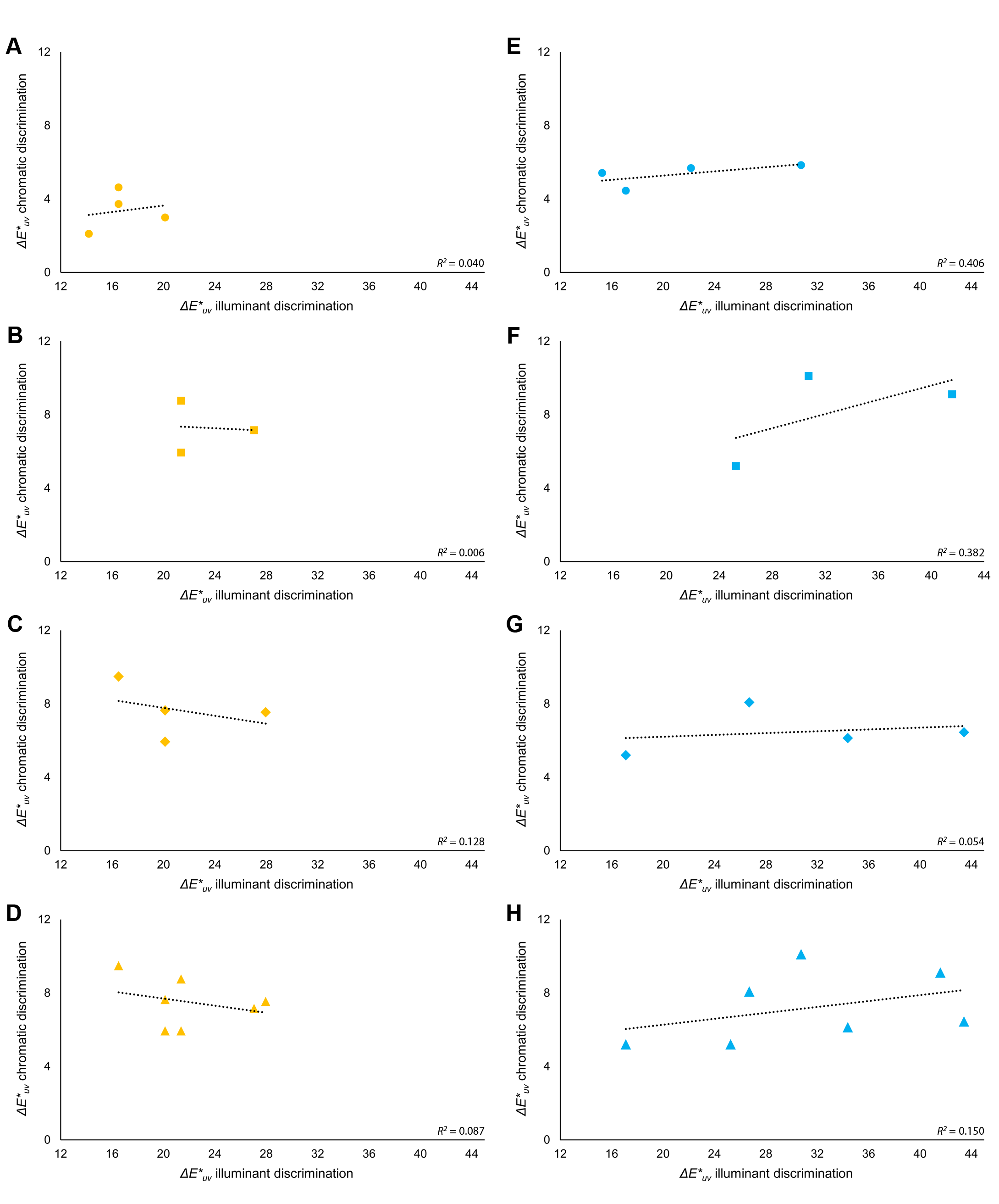

Supplement: S1 Fig — Colour discrimination thresholds in ΔE*uv along daylight locus (y-axis) for the two illumination directions in Fig 2 (towards yellowish illuminants, panels A-D; towards bluish illuminants, panels E-H) in relation with the reference white (u´, = 0.1947; v´ = 0.4639; Y = 11 cd/m2), for normal observers (panels A and E, solid circles), protanopes (panels B and F, squares), deuteranopes (panels C and G, diamonds) and both dichromat groups collapsed (panels D and H, triangles) against illuminant discrimination thresholds in ΔE*uv along daylight locus (x-axis) for the two illumination change directions of the CCT condition (towards yellowish illuminants, panels A-D; towards bluish illuminants, panels E-H) of experiment 1 in relation with the reference illuminant (u´, = 0.1968; v´ = 0.4663; Y = 10 cd/m2). R2 (all p>.05) and the best-fitting lines derived from linear least-squares regressions are also given. (TIF) [file pone.0180310.s002.tif]
