# Supplementary material for: Robust colour constancy in red-green dichromats
Source: PLoS One. 2017 Jun 29;12(6):e0180310. doi: 10.1371/journal.pone.0180310 (PMC5491172; doi:10.1371/journal.pone.0180310)
Supplement: S1 Dataset — (PDF) [file pone.0180310.s003.pdf]

Experiment 1

Diagnosis	Id	CCT_Im1_Down	CCT_Im1_Up	CCT_Im2_Down	CCT_Im2_Up	CCT_Im3_Down	CCT_Im3_Up	CCT_Im4_Down	CCT_Im4_Up
3	N1	199.25	124.25	174.25	99.25	174.25	124.25	174.25	124.25
3	N2	174.25	99.25	174.25	99.25	174.25	99.25	174.25	124.25
3	N3	174.25	124.25	174.25	124.25	174.25	124.25	174.25	124.25
3	N4	174.25	124.25	174.25	124.25	174.25	124.25	160.79	135.61
1	P1	174.25	99.25	199.25	99.25	160.79	124.25	199.25	99.25
1	P2	174.25	74.25	174.25	74.25	224.25	99.25	199.25	124.25
1	P3	199.25	99.25	174.25	99.25	199.25	124.25	160.79	135.61
2	D1	174.25	124.25	199.25	99.25	199.25	124.25	199.25	99.25
2	D2	174.25	124.25	199.25	124.25	174.25	124.25	174.25	124.25
2	D3	174.25	124.25	174.25	49.25	174.25	99.25	174.25	124.25
2	D4	174.25	99.25	174.25	99.25	199.25	99.25	174.25	99.25

Diagnosis	Id	Lum_Im1_Down	Lum_Im1_Up	Lum_Im2_Down	Lum_Im2_Up	Lum_Im3_Down	Lum_Im3_Up	Lum_Im4_Down	Lum_Im4_Up
3	N1	6.00	13.00	7.00	13.00	7.00	13.00	7.00	11.00
3	N2	7.00	12.00	9.00	15.00	8.00	12.00	8.00	13.00
3	N3	7.00	12.00	8.00	15.00	7.00	13.00	8.00	13.00
3	N4	7.00	14.00	8.00	13.00	7.00	14.00	8.00	14.00
1	P1	6.00	15.00	8.00	15.00	6.00	15.00	7.00	15.00
1	P2	6.00	13.00	7.00	14.00	7.00	15.00	7.00	15.00
1	P3	7.00	12.00	6.00	13.00	6.00	13.00	7.00	13.00
2	D1	7.00	13.00	7.00	13.00	8.00	13.00	8.00	12.00
2	D2	7.00	12.00	8.00	12.00	7.00	13.00	7.00	13.00
2	D3	7.00	12.00	7.00	14.00	8.00	14.00	8.00	14.00
2	D4	7.00	15.00	7.00	15.00	6.00	14.00	6.00	15.00

Diagnosis	Id	IncE_Down	IncE_Up	IncE_Ydiscrim	IncE_Bdiscrim
3	N1	20.13	22.18	2.99	5.68
3	N2	16.51	30.75	4.63	5.84
3	N3	16.51	17.10	3.73	4.45
3	N4	14.19	15.26	2.10	5.42
1	P1	21.37	30.75	8.76	10.11
1	P2	27.05	41.58	7.16	9.11
1	P3	21.37	25.26	5.94	5.20
2	D1	27.96	26.71	7.54	8.08
2	D2	20.13	17.10	5.94	5.20
2	D3	16.51	43.43	9.49	6.45
2	D4	20.13	34.38	7.64	6.13

Note. N, P and D stand for Normal, Protanope and Deuteranope
