# Supplementary material for: Robust colour constancy in red-green dichromats
Source: PLoS One. 2017 Jun 29;12(6):e0180310. doi: 10.1371/journal.pone.0180310 (PMC5491172; doi:10.1371/journal.pone.0180310)
Supplement: S2 Dataset — (PDF) [file pone.0180310.s004.pdf]

Experiment 2

Diagnosis	Id	CCT_Im1_Up	CCT_Im1_Down	CCT_Im2_Up	CCT_Im2_Down	CCT_Im3_Up	CCT_Im3_Down	CCT_Im4_Up	CCT_Im4_Down
3	N1	1.00	1.00	1.00	1.00	1.00	1.00	1.00	1.00
3	N2	1.00	1.00	1.00	1.00	1.00	0.90	0.90	1.00
3	N3	0.70	0.50	0.80	0.30	0.70	0.90	0.40	0.70
3	N4	0.30	1.00	1.00	1.00	0.90	0.90	0.90	1.00
1	P1	0.10	1.00	0.00	1.00	1.00	1.00	1.00	1.00
1	P2	1.00	0.90	0.00	0.70	0.60	1.00	0.90	0.90
1	P3	1.00	1.00	0.30	1.00	0.40	1.00	0.20	1.00
2	D1	1.00	1.00	0.90	1.00	1.00	1.00	1.00	1.00
2	D2	1.00	1.00	0.80	0.90	1.00	1.00	1.00	1.00
2	D3	0.50	0.90	0.90	0.20	0.60	1.00	0.10	0.40
2	D4	1.00	1.00	0.00	1.00	1.00	1.00	1.00	1.00

Diagnosis	Id	Lum_Im1_Up	Lum_Im1_Down	Lum_Im2_Up	Lum_Im2_Down	Lum_Im3_Up	Lum_Im3_Down	Lum_Im4_Up	Lum_Im4_Down
3	N1	1.00	1.00	1.00	1.00	1.00	1.00	1.00	1.00
3	N2	1.00	1.00	1.00	1.00	1.00	0.90	1.00	1.00
3	N3	0.70	0.50	0.60	0.70	0.70	0.90	0.60	0.70
3	N4	1.00	1.00	0.90	0.90	1.00	1.00	1.00	1.00
1	P1	0.80	0.10	0.30	0.10	0.30	0.70	0.20	0.90
1	P2	1.00	0.90	1.00	0.90	1.00	0.80	0.80	0.50
1	P3	1.00	1.00	1.00	1.00	1.00	1.00	1.00	1.00
2	D1	1.00	1.00	1.00	1.00	1.00	1.00	1.00	0.90
2	D2	0.90	1.00	1.00	1.00	1.00	1.00	1.00	1.00
2	D3	1.00	0.00	1.00	0.00	0.90	0.00	1.00	0.00
2	D4	1.00	0.80	0.60	0.80	1.00	0.90	0.80	0.90

Note. N, P and D stand for Normal, Protanope and Deuteranope
